# Caveolae Restrict Tiger Frog Virus Release in HepG2 cells and Caveolae-Associated Proteins Incorporated into Virus Particles

**DOI:** 10.1038/srep21663

**Published:** 2016-02-18

**Authors:** Jian He, Yi-Wen Zheng, Yi-Fan Lin, Shu Mi, Xiao-Wei Qin, Shao-Ping Weng, Jian-Guo He, Chang-Jun Guo

**Affiliations:** 1 Guangdong Provincial Key Laboratory of Marine Resources and Coastal Engineering/South China Sea Bio-Resource Exploitation and Utilization Collaborative Innovation Center, School of Marine, Sun Yat-sen University, 135 Xingang Road West, Guangzhou 510275, PR China; 2 MOE Key Laboratory of Aquatic Product Safety/State Key Laboratory for Biocontrol, School of Life Sciences, Sun Yat-sen University, 135 Xingang Road West, Guangzhou 510275, PR China; 3 Institute of Aquatic Economic Animals and Guangdong Province Key Laboratory for Aquatic Economic Animals, Sun Yat-sen University, 135 Xingang Road West, Guangzhou 510275, PR China

## Abstract

Caveolae are flask-shaped invaginations of the plasma membrane. Caveolae play important roles in the process of viruses entry into host cells, but the roles of caveolae at the late stage of virus infection were not completely understood. Tiger frog virus (TFV) has been isolated from the diseased tadpoles of the frog, *Rana tigrina rugulosa*, and causes high mortality of tiger frog tadpoles cultured in Southern China. In the present study, the roles of caveolae at the late stage of TFV infection were investigated. We showed that TFV virions were localized with the caveolae at the late stage of infection in HepG2 cells. Disruption of caveolae by methyl-β-cyclodextrin/nystatin or knockdown of caveolin-1 significantly increase the release of TFV. Moreover, the interaction between caveolin-1 and TFV major capsid protein was detected by co-immunoprecipitation. Those results suggested that caveolae restricted TFV release from the HepG2 cells. Caveolae-associated proteins (caveolin-1, caveolin-2, cavin-1, and cavin-2) were selectively incorporated into TFV virions. Different combinations of proteolytic and/or detergent treatments with virions showed that caveolae-associated proteins were located in viral capsid of TFV virons. Taken together, caveolae might be a restriction factor that affects virus release and caveolae-associated proteins were incorporated in TFV virions.

Caveolae are flask-shaped invaginations of the cell plasma membrane with diameters in the range of 50–100 nm^1^. Caveolae are found in numerous cell types and are especially abundant in adipocytes, myocytes, and endothelia^2^. Caveolae are a subset of lipid rafts that have specific morphology and composition, which distinguish caveolae membranes from other lipid rafts^3^. Cholesterol is a major component of caveolae, and depletion of cellular cholesterol, either by extraction using methyl-β-cyclodextrin (MβCD) or sequestration of cholesterol with nystatin, reduces the number of invaginated caveolae^4^,^5^. Caveolin-1, caveolin-2, and caveolin-3 proteins are three key structural components of caveolae. Caveolin-1, the principal structural component of caveolar membranes, is involved in a wide range of cellular processes, such as cell cycle regulation, signal transduction, transcytosis, endocytosis, cholesterol homeostasis, and apoptosis^6^,^7^,^8^,^9^. Ablation of caveolin-1 causes caveolae loss^10^. Caveolin-1 interacts directly with caveolin-2, thereby forming a heteroligomeric complex with high molecular mass that targets lipid rafts and drives the formation of caveolar structures^11^,^12^. Caveolin-1 and caveolin-2 are co-expressed ubiquitously, whereas caveolin-3 is a muscle-specific isoform that functionally substitutes for caveolin-1 in skeletal and heart muscle cells^13^. Cavins are essential caveolae-associated proteins and are important for caveolae biogenesis. Cavins have differential tissue distributions. Four different cavin proteins have been identified, as follows: cavin-1 (PTRF, polymerase I and transcript release factor), cavin-2 (SDPR, serum deprivation protein response), cavin-3 (SRBC, sdr-related gene product that binds to C-kinase), and cavin-4 (MURC, muscle-restricted coiled-coil protein)^14^. Cavins regulate caveolar function and organization, and each cavin is assigned different roles based on caveolar morphology and cell type^15^. Cavin proteins function primarily as scaffolding and also regulate caveolin availability^16^. An increasing evidence shows that several pathogens, such as viruses, bacteria, and parasites, use caveolae for their own benefit^17^. On one hand, some viruses, such as HIV, SV40 (simian virus 40), RSV (respiratory syncytial virus), and echovirus 1, use caveolae to enter the host^18^,^19^,^20^,^21^. On the other hand, raft domains provide assembly and budding sites for viruses, such as influenza virus, measles virus, rotavirus, and herpes simplex virus^22^,^23^,^24^,^25^. Caveolae are a subset of lipid rafts, but the roles of caveolae at the late stage of virus infection not completely understood.

Iridoviruses are large, icosahedral cytoplasmic DNA viruses that contain circularly permutated, terminally redundant, double-stranded DNA genomes^26^,^27^. Iridoviruses cause systemic diseases in invertebrates, particularly insects and poikilothermic vertebrates (fish, amphibians and reptiles)^28^. To date, more than 100 iridovirus strains have been isolated, and entire genomes of more than 20 strains have been completely sequenced^28^. The *Iridoviridae* family is composed of five genera, as follows: *Iridovirus*, *Chloriridovirus*, *Ranavirus*, *Lymphocystivirus*, and *Megalocytivirus*^29^. The first reported complete genome sequence in the genus *Ranavirus* is tiger frog virus (TFV). TFV is isolated from the infected tadpoles of *Rana tigrina rugulosa*, causes high mortality rate among tiger frog tadpoles cultured in Southern China^30^. Recent reports showed that caveolae participate in the invasion of some iridoviruses. Infectious spleen and kidney necrosis virus (a megalocytivirus) enters mandarin fish fry (MFF-1) cells through a caveolae-dependent endocytic pathway and may colocalize with caveolin-1^31^. Research on the uptake of TFV into HepG2 cells at 27 °C show a pH-dependent, atypical caveolae-dependent endocytosis via the acidification of intracellular organelles^32^. In the present study, the roles of caveolae at the late stage of virus infection are explored, and the major structural proteins of caveolae are identified incorporated into TFV virions.

## Results

### TFV virions localized with caveolae/caveolar endocytotic vesicle at the late stage of infection

**Figure 1:**
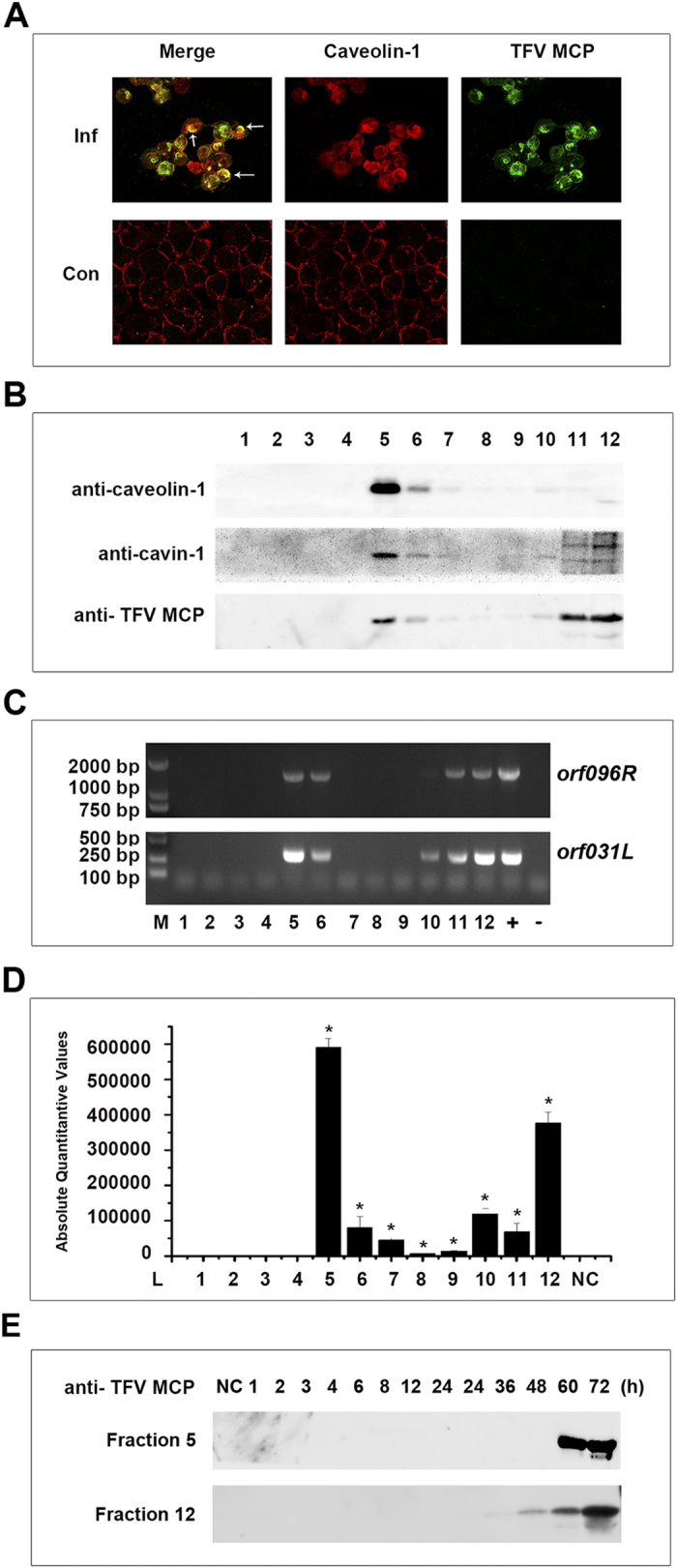
Caveolin-1/caveolae/caveolar endocytotic vesicle colocalized with TFV virions at the late phase of infection. (**A**) HepG2 cells grown on coverslips were either uninfected (Con.) or infected (Inf.) by TFV at an MOI of 10. The cells were fixed with paraformaldehyde at 72 h postinfection. Caveolin-1 and TFV MCP were stained as described in the Materials and Methods. Bound IgG was detected with host-specific secondary antibodies conjugated to either Alexa Fluor 488 or 555. The yellow overlay represents colocalization of TFV MCP and caveolin-1 (white arrows). (**B**) HepG2 cells infected with TFV after 72 h postinfection. Cells were extracted with 1% Triton X-100 at 4 °C. The lysate was loaded at the bottom of a flotation sucrose density gradient and subjected to equilibrium centrifugation. The gradient was fractionated from the top, and polypeptides were analyzed by SDS-PAGE and immunoblotting. (**C**) Extracted DNA from each fractions were amplified TFV gene *orf031L* and *orf096R* by PCR, then detected by nucleic acid electrophoresis experiment (+ indicates positive control,- indicates negative control). (**D**) Extracted DNA from each fractions and then detected TFV gene *orf096R* by absolute quantitative real-time PCR. Y-axe represent absolute quantitative values of TFV gene *orf096R*. Asterisks indicate values that are statistically significant (p < 0.05) compared with values for NC. (**E**) Caveolae were isolated on sucrose gradients at different time points from 1 to 72 h post-infection, and equal volume fractions were separated. The fractions 5 and 12 of each time points were detected with anti-TFV MCP antibody by SDS-PAGE and immunoblotting.

Dual-color fluorescence staining was used to prove the relationship between caveolae and TFV virions at 72 h postinfection. Caveolin-1 was used as the marker of caveolae or caveolar endocytotic vesicle. Caveolin-1 demonstrated punctuate staining pattern that is mainly localized in the plasma membrane of uninfected HepG2 cells (Fig.1A, Con, red fluorescence), which is consistent with its known localization in clustered caveolae microdomains^33^. After infecting with TFV, staining for TFV major capsid protein (MCP) showed that the protein was concentrated in patches in the cytoplasm of the virus-infected cells (Fig.1A, Inf, green fluorescence). The inset in Fig. 1A (yellow fluorescence, arrowed) shows that the punctate pattern caveolin-1 staining localized in the patches containing MCP stain, thereby suggesting that TFV MCP localizes with caveolin-1/caveolae/caveolar endocytotic vesicle at the late stage of infection.

Caveolae and raft membrane microdomains, enriched with sphingolipids and cholesterol can be isolated on low-density sucrose gradients by their insolubility in Triton X-100 at 4 °C^34^,^35^. The caveolae were isolated on low-density sucrose gradients 72 h postinfection, and equal volume fractions were separated using SDS-PAGE and immunoblotting. As shown in Fig. 1B, caveolin-1 and cavin-1 were highly enriched in fractions 5 and 6, which corresponded to the interphase between 5% and 30% sucrose, and those fractionations pattern are consistent with the known sedimentation properties of caveolae membranes^36^,^37^. In addition, TFV MCP floated into the caveolae-rich fractions (Fig. 1B), indicating that TFV MCP localized in caveolae at the late phase of infection. To further confirm the existence of TFV virions, viral genome DNA was extracted from each fraction and detected TFV *orf031L* gene and *mcp* gene (*orf96R*) using PCR and qPCR. TFV *orf031L* and *mcp* gene were determined in fractions 5 and 6 (Fig. 1C). The highest expression level of TFV *mcp* gene was observed in fraction 5 (Fig.1D). Together, these results indicated that TFV virions localized in caveolae at the late phase of infection.

To investigate the profile of caveolae colocalization with TFV during virus infection, caveolae were isolated on sucrose gradients at different time points from 1 h to 72 h post-infection, and equal volume fractions were separated. The fractions 5 and 12 were detected by using SDS-PAGE and western blotting, since the fractions 5 were consistent with the known sedimentation properties of caveolae membranes while fraction 12 were with cytoplasm. As shown in Fig.1E, TFV MCP started to be detected at 36 h postinfection and the signal became stronger gradually at 48, 60 and 72 h in fractions 12, suggesting that TFV MCP begins to synthesis at 36 h postinfection in HepG2 cells. However, TFV MCP could not be detected in fraction 5 until 60 h postinfection, suggesting that TFV MCP colocalize with caveolae after 60 h postinfection. The results suggested that TFV colocalize with caveolae started at 60 h postinfection, indicating TFV detected in caveolae represent newly formed viruses but not the ones have entered inside the host cells.

### Caveolae restricted the release of TFV virions

**Figure 2:**
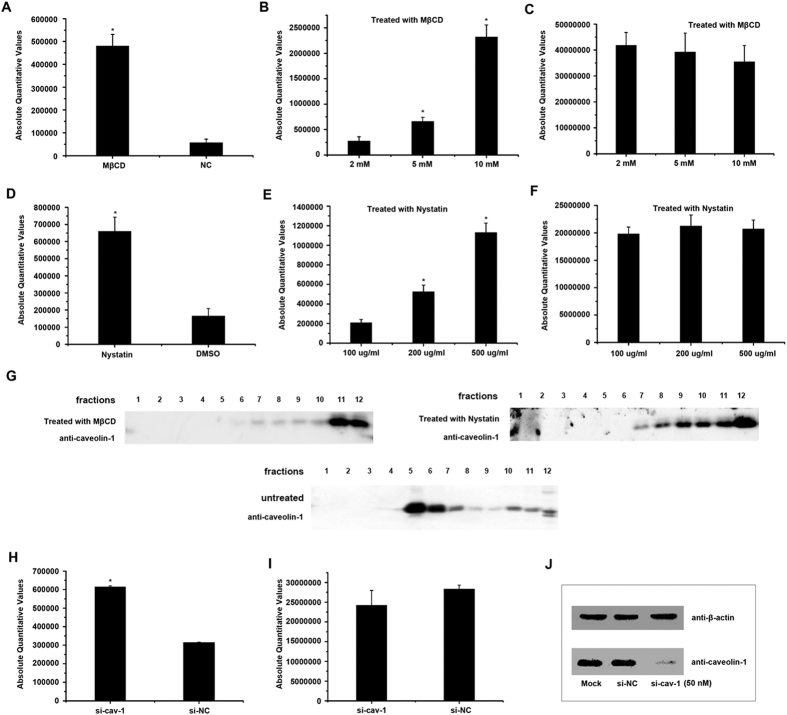
Caveolae inhibit the release of TFV virions. 60 h after HepG2 cells were infected with TFV at an MOI of 10, the cells were treated with 2, 5 and 10 mM MβCD, or 100, 200 and 500 μg/ml nystatin for 12 h. Y-axes represent absolute quantitative values of TFV gene *orf096R*. Asterisks indicate values that are statistically significant (p < 0.05). (**C**,**F**) The absolute amount of TFV *mcp* gene *orf96R* from the infected cells at the different concentrations of MβCD or nystatin were tested. (**A**,**D**) The absolute amount of TFV *mcp* gene *orf96R* from the supernatant of the cells was determined by qtPCR after MβCD (5 mM) or nystatin (200 μg/ml) treatments. Cells not treated with MβCD or cells treated with DMSO respectively as controls. (**B**,**E**) With increasing dose of MβCD or nystatin, the amount of TFV *mcp* gene *orf96R* was measured. (**G**) After MβCD (5 mM) or nystatin (200 μg/ml) treatments. Cells were extracted with 1% Triton X-100 at 4 °C. The lysate was loaded at the bottom of a flotation sucrose density gradient and subjected to equilibrium centrifugation. The gradient was fractionated from the top, and polypeptides were analyzed by SDS-PAGE and immunoblotting. (**H**) The amount of TFV *mcp* gene *orf96R* in supernatant was measured after transfected with 50 nm si-caveolin-1 or 50 nm si-NC. (**I**) The absolute amount of TFV *mcp* gene *orf96R* from the infected cells transfected with 50 nm si-caveolin-1or 50 nm si-NC. (**J**) Si-caveolin-1 was used to disrupt caveolae as described above. The most effective concentration was 50 nM.

**Figure 3:**
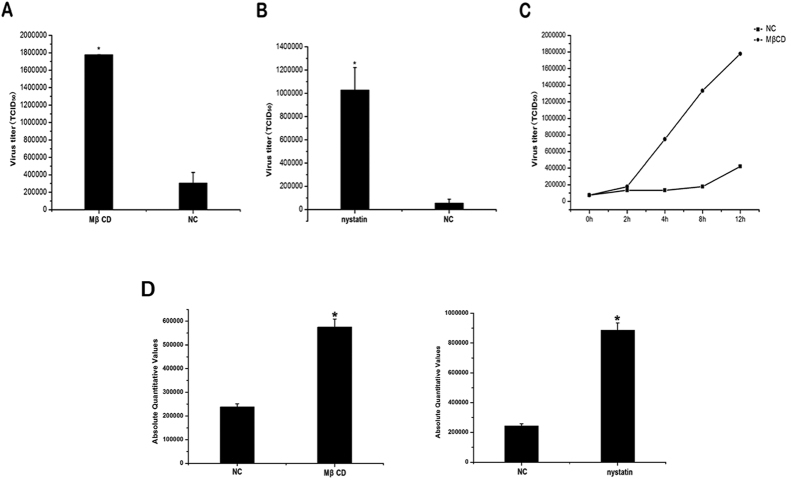
Virus titers of FHM cells infected with TFV. 60 h after HepG2 cells were infected with TFV at an MOI of 10. (**A**) The cells were treated with 5mM MβCD, or (**B**) 200 μg/ml nystatin for 12 h. The supernatant of the cells was collected and the Virus titers were measured. (**C**) 60 h after HepG2 cells were infected with TFV at an MOI of 10, the cells were treated with 5 mM MβCD or not (NC). The supernatant of the cells was collected at different time point (0, 2, 4, 8 and 12 h) after treated and the Virus titers were measured. Y-axes represent Virus titers. (**D**) 60 h after FHM cells were infected with TFV at an MOI of 0.1, the cells were treated with 2 mM MβCD, or 100 μg/ml nystatin for 12 h. The supernatant of the cells was collected and the virus titers were measured. Y-axes represent absolute quantitative values of TFV gene *orf096r*. Asterisks indicate values that are statistically significant (p < 0.05).

Depletion of cholesterol from membranes with MβCD or sequestration of cholesterol with nystatin impairs caveolae-mediated endocytosis^38^. To investigate the roles of caveolae at the late stage of TFV infection, HepG2 cells were treated with 5 mM MβCD or 200 μg/ml nystatin at 60 h postinfection. After HepG2 cells were treated with MβCD or nystatin, caveolae were isolated on sucrose gradients using the above methods. As the results shown in Fig. 2G, caveolin-1 was not detected in the fractions 5 but in fractions 11 and 12 (cytoplasmic fragment), which suggested that caveolae were depolymerized after treated with MβCD or nystatin. TFV *mcp* gene from the supernatant of cells at 72 h postinfection was quantified using qPCR. As shown in Fig. 2A,D, the amount of TFV virions in the supernatant of treated-HepG2 cells increased. When the dose of MβCD or nystatin increased, the amount of TFV correspondingly increased in a dose-dependent manner (Fig. 2B,E). The absolute amount of TFV *mcp* gene from the infected cells had no significant change after treated with the different concentrations of MβCD or nystatin (Fig. 2C,F). This result showed that the concentrations of the chemicals used in this study did not affect TFV replication. The virus titers assay was used to measure the infectious virions that released from cell into the supernatant. HepG2 cells were treated with 5 mM MβCD or 200 μg/ml nystatin at 60 h postinfection. The supernatant of cells at 72 h postinfection was collected and the TCID_50_ was measured for each sample. As shown in Fig. 3A,B, the virus titer of TFV in the supernatant of treated-HepG2 cells increased. The single step growth curves (Fig. 3C) showed that infectious virions in the supernatant increased after cells treated with 5 mM MβCD. Caveolin-1 is the principal organizer of caveolae. Knockdown of caveolin-1 impairs the formation of caveolae^39^. In our study, si-caveolin-1 was used to knockdown the gene to detect the level of TFV release. The most effective si-caveolin-1 concentration is 50 nM (Fig. 2J), and TFV replication was not affected at this concentration (Fig. 2I). However, the amount of TFV in the supernatant was higher with the si-caveolin-1 transfection than with the si-NC control (Fig. 2H). Therefore, these data indicated that caveolae restricted TFV release from HepG2 cells.

To explore whether caveolae can restrict TFV release from more relevant cell lines. FHM cells were infected with TFV and were then treated with MβCD or nystatin at 60 h postinfection. Control cells were incubated in medium with the corresponding solvent. The TFV *mcp* gene from the supernatant of cells at 72 h postinfection was quantified using qPCR. As shown in Fig. 3D, the amount of TFV virions in the supernatant of treated-FHM cells increased. The results suggested that caveolae might also restrict TFV release from FHM cells.

### Caveolin-1 interacts with TFV MCP

**Figure 4:**
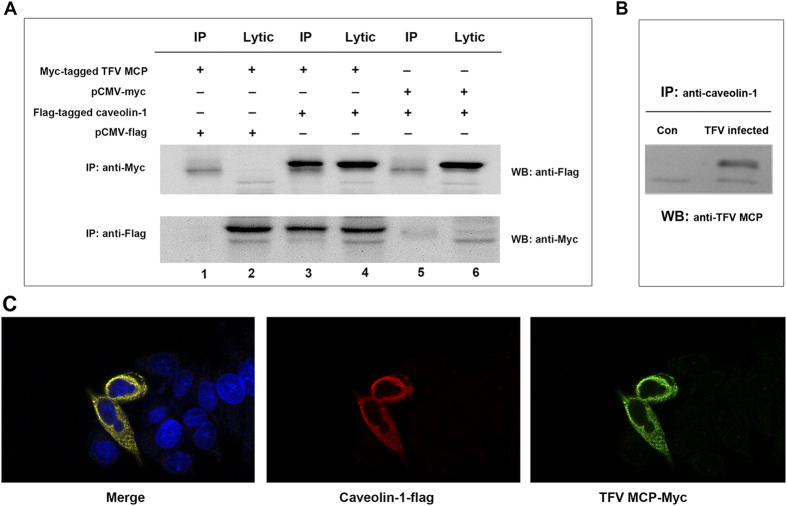
Caveolin-1 interact with TFV MCP. (**A**) CO-IP assay. HeLa cells were transiently transfected with myc-tagged TFV MCP and pCMV-flag empty vector (lanes 1 and 2), Flag-tagged caveolin-1 protein and myc-tagged TFV MCP, (lanes 3 and 4), pCMV-myc empty vector and FLAG-tagged caveolin-1 protein (lanes 5 and 6), respectively, as indicated (lane 3). Immunoprecipitation (IP) of Myc-tagged TFV MCP with Myc tag-specific monoclonal antibody led to coprecipitation of Flag-tagged caveolin-1 protein. IP of caveolin-1 protein Flag-tagged using Flag-tag-specific monoclonal resulted in coprecipitation of Myc-tagged TFV MCP. (**B**) HepG2 cells were infected with TFV at an MOI of 10. At 72 h postinfection, we collected the cells to do the immunoprecipitation using anti-caveolin-1 antibody (TFV infected), uninfected HepG2 cells as control (con). (**C**) Colocalization of caveolin-1 with TFV MCP was examined in HeLa cells transiently expressing Flag-tagged caveolin-1 protein and Myc-tagged TFV MCP. The intracellular localization of TFV MCP and caveolin-1 was analyzed by IFA using anti-Flag and anti-Myc antibodies. Caveolin-1 (red fluorescence) and TFV MCP (green fluorescencce) were viewed under a confocal microscope equipped with 555/488 nm argon-krypton and 543 nm helium-neon lasers. The yellow overlay represents colocalization of TFV MCP and caveolin-1. All nuclei were stained with a blue fluorescent dye (Hoechst 33342).

To identify the caveolin-1 interaction with TFV protein, the interaction between human caveolin-1 and TFV MCP was observed using Co-IP experiments. HeLa cells were transiently transfected to produce Flag-tagged caveolin-1 protein and myc-tagged TFV MCP. Immunopecipitation of myc-tagged TFV MCP by using myc tag-specific monoclonal antibody led to coprecipitation of Flag-tagged caveolin-1 protein (Fig. 4A, lane 3). In a converse experiment, immunoprecipitation of Flag-tagged caveolin-1 protein using Flag-tag specific monoclonal resulted in coprecipitation of myc-tagged TFV MCP (Fig. 4A, lane 3). The results were further supported by colocalization of caveolin-1 with TFV MCP found in HeLa cells transiently expressing Flag-tagged caveolin-1 protein and myc-tagged TFV MCP (Fig. 4C). The results proved that caveolin-1 physically interacted with TFV MCP and suggested the mechanism underlying the restriction of TFV by caveolae might be via the interaction of caveolin-1 and TFV MCP.

To corroborate interaction between caveolin-1 and TFV MCP under non-over expression conditions. The immunoprecipitation experiments were carried out with the antibodies against caveolin-1 in the cells infected with virus. HepG2 cells were infected with TFV at an MOI of 10. At 72 h postinfection, we collected the cells to do the immunoprecipitation using anti-caveolin-1 antibody with uninfected HepG2 cells as control. As shown in Fig. 4B, TFV MCP can be immunoprecipitated by anti-caveolin-1 antibody, suggesting that TFV MCP interacts with caveolin-1 at the late stage of virus infection.

### Caveolae-associated proteins were incorporated into TFV virions

**Figure 5:**
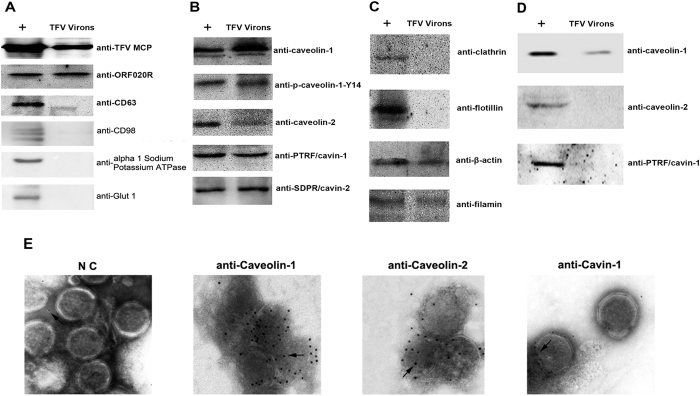
Caveolae-associated proteins were incorporated in TFV virions. Purified TFV through sedimentation in sucrose density gradients from supernatant of TFV infected HepG2 cell lines. Whole-cell lysates from the TFV-infected HepG2 (+ indicate positive control) and TFV virions underwent immunoblotting. (**A**) The blots presented in panel A were detected by viral structural proteins (MCP, ORF020R) and host proteins (CD63, CD98, alpha 1 Sodium Potassium ATPase, Glut-1). (**B**) The blots presented in panel B were detected by anti-caveolin-1, anti-caveolin-2, anti-PTRF/cavin-1, and anti-SDPR/cavin-2. (**C**) The blots presented in panel C were detected by anti-clathrin, anti-flotillin, anti-β-actin, and anti-filamin. (**D**) MβCD changed the incorporation of caveolar components. Whole-cell lysates from the TFV-infected HepG2 (+ indicate positive control) and TFV virions underwent immunoblotting. The blots presented were detected by anti-caveolin-1, anti-caveolin-2, anti-PTRF/cavin-1. (**E**) Purified TFV virions were labeled by anti-caveolin-1, anti-caveolin-2 and anti-PTRF/cavin-1 after which goat anti-rabbit IgG conjugated with 10 nm colloid gold was used for labeling the primary antibody. The NC used Negative rabbit serum as the primary antibody.

HepG2 cells became round and yielded TFV virions at 6 d postinfection with TFV at an MOI of 10. The supernatant was harvested to purify TFV by sedimentation in sucrose density gradients. Western blot analysis results showed that the viral envelope protein vp020R and the viral MCP were detected in our purified virus sample, but not CD63 (a marker of microvesicles; used as control), CD98, alpha 1 Sodium Potassium ATPase and Glut-1 (markers of plasma membrane; used as control) (Fig. 5A). Those results showed that the TFV used in the experiment was without microvesicles or cell debris. To explore whether caveolae-associated proteins were incorporated into TFV virions, the major structural proteins of caveolae and the cytoskeletal elements were detected by western blot. The major structural proteins (caveolin-1, caveolin-2, cavin-1, and cavin-2) were present in the TFV virions (Fig. 5B). To provide additional evidence for the incorporation of caveolae-associated proteins in TFV virions, immunogold labeling was performed. As shown in Fig. 5E, several gold particles were labeled on the virions using anti-caveolin-1, anti-caveolin-2 or anti-cavin-1 antibody as the primary antibody. Cytoskeletal elements, such as β-actin and filamin, were detected in the purified TFV virions (Fig. 5C). Interestingly, the phosphorylation form of caveolin-1 (p-caveolin-1-Y14) was also detected in TFV virions (Fig. 5B). Other lipid raft proteins, namely, clathrin and flotillin, were not detected in purified TFV virions (Fig. 5C). These observations suggested that caveolae-associated proteins were incorporated into TFV virions.

To test whether depolymerized caveolae affected the released virions in terms of incorporation of caveolar components. HepG2 cells were infected with TFV at an MOI of 10 and at 60 h postinfection were treated with 5 mM MβCD. The supernatant was harvested 12 h after treating with MβCD. TFV virions were purified by sedimentation in sucrose density gradients. Western blot analysis results showed that only caveolin-1 can be detected but not caveolin-2 and cavin-1 (Fig. 5D), suggesting that treating with MβCD changed the incorporation of caveolar components.

### Caveolin-1 was selectively incorporated into virions

**Figure 6:**
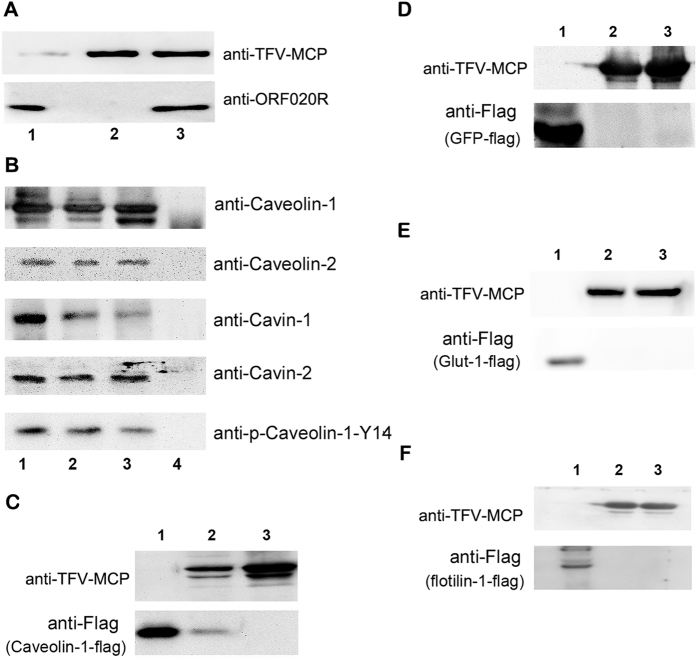
The location of caveolae-associated proteins in TFV virions and selective incorporation of caveolin-1 in TFV virions. (**A**) Purified virions were treated with 0.5% Triton X-100 for 10 min and were centrifuged at 20000 rpm. The supernatant (lane 1) and precipitation (lane 2) underwent immunoblotting for viral proteins (MCP, ORF020R). Purified virions (lane 3) were used as a positive control. (**B**) Purified virions were subjected to different combinations of proteolytic and detergent treatments. The results of these treatments were observed from the envelope to the capsid. The resulting particles underwent gradient purification and analysis by immunoblotting. Untreated purified TFV virions contained caveolin-1 (lane 1). Potential caveolin-1 outside the envelope (lane 2) was removed by subtilisin digestion. Purified virions were treated with 0.5% Triton X-100 for 10 min (lane 3). After treated with 0.5% Triton X-100 for 10 min, the naked virions were digested by subtilisin (lane 4). Treated virions were analyzed by using anti-caveolin-1, anti-p-caveolin-1-Y14, anti-caveolin-2, anti-PTRF/cavin-1, or anti-SDPR/cavin-2 antibody. HepG2 cells were transfected to express caveolin-1 with Flag-tag (**C**, lane 1), GFP with Flag-tag (**D**, lane 1), Glut-1 with Flag-tag (**E**, lane 1) and flotillin-1 (**F**, lane 1) with flag-tag. as a positive control for caveolin-1-flag, GFP-Flag, Glut-1-Flag and flotillin-1. TFV virions (**C**–**F** lanes 3) were used as a positive control for TFV MCP. HepG2 cells were transfected to express caveolin-1-Flag, GFP-Flag, Glut-1-Flag or flotillin-1-flag. and the cells were infected with TFV 24 h posttransfection (p.i.). The supernatant was harvested to purify TFV by sedimentation in sucrose density gradients at 6 d postinfection and analyzed by SDS-PAGE and immunoblotting (**C–F** lanes 2).

To confirm that caveolin-1 was selectively incorporated into TFV virions, we tested whether the exogenous fusion caveolin-1-flag could also be incorporated into the TFV virions. HepG2 cells were transfected to express caveolin-1 with flag-tag, and the control cells were transfected to express green-fluorescent protein (GFP), Glut-1 (a membrane protein) or flotillin-1 (a lipid raft microdomain-associated protein) with flag-tag. The cells were then infected with TFV at 24 h post-transfection and the supernatant was harvested to purify TFV by sedimentation in sucrose density gradients at 6 d postinfection. As shown in Fig. 6C, caveolin-1 was detected by flag-tag specific monoclonal antibody. However, GFP (Fig. 6D), Glut-1 (Fig. 6E) and flotillin-1 (Fig. 6F) proteins were not detected in purified virions. Those results suggested that caveolin-1 was selectively incorporated into TFV virions.

Ranavirus particles consist of an inner DNA/protein core, an internal limiting membrane, a viral capsid, and an outer viral envelope^29^. To explore the location of caveolae-associated proteins within TFV virions, virions were subjected to different combinations of proteolytic and/or detergent treatments from the envelope to the capsids, after which gradient purification of the resulting particles and western blot analysis were performed. ORF020R is a TFV envelope protein^40^. As shown in Fig. 6A, after treating with 0.5% Triton X-100 for 10 min, purified TFV virions were centrifuged at 20000 rpm. ORF020R could be detected in supernatant (Fig. 6A, lane 1), but not in the precipitation (Fig. 6A, lane 2). The results proved that the outer viral envelope of TFV were removed by Triton X-100.

Untreated purified TFV virions incorporated caveolin-1, p-caveolin-1-Y14, caveolin-2, cavin-1, and cavin-2 proteins (Fig. 6B, lane 1) and were used as positive controls. Proteins outside the envelope were removed (Fig. 6B, lane 2) by subtilisin digestion. To degrade the envelope, virions were treated with 0.5% Triton X-100 for 10 min (Fig. 6B, lane 3). The signal persisted until the naked virions were digested by subtilisin (Fig. 6B, lane 4). Taken together, these observations suggest that caveolae-associated proteins (caveolin-1, p-caveolin-1-Y14, caveolin-2, cavin-1, and cavin-2) were incorporated in the TFV virions and located in viral capsid of TFV virion.

## Discussion

In the present study, we found that caveolae restrict TFV release and caveolae-associated proteins were incorporated into TFV particles in HepG2 cells. Virions localization with caveolae were observed at the last stages of infection. Disruption of caveolae by MβCD/nystatin or knockdown caveolin-1 increased the release of the virus, suggesting that caveolae play important roles at the late stages of viral life cycle and may restrict virus release. The mechanism underlying the restriction may be via the interaction of caveolin-1 and TFV MCP. Moreover, the major structural proteins of caveolae (caveolin-1, caveolin-2, cavin-1, and cavin-2) were selectively incorporated into the mature virions, and were present in the capsid of TFV virions.

Caveolae can be used by viruses for their own benefit. Caveola-dependent endocytosis was originally identified as an entry pathway for SV40^21^. Since then, the use of caveolae for entry into host cells has been demonstrated for other viruses, such as HIV, RSV, and echovirus 1^18^,^19^,^20^,^21^. In this study, numerous TFV virions were localized in caveolae/caveolar endocytotic vesicle at 72 h postinfected (Fig. 1A–C). The time course experiment showed that TFV colocalized with caveolae started at 60 h postinfection (Fig. 1E), indicating that TFV detected in caveolae represent newly formed viruses but not the ones have entered inside the host cells. Those results suggested that caveolae may take part in the late stages of TFV life cycle.

To investigate the function of caveolae at the late phase of TFV life cycle, we disrupted the caveolae using MβCD and nystatin, and found that the amount of TFV virions in the supernatant increased (Figs 2 and 3). We used siRNA knockdown caveolin-1 to impair the formation of caveolae and acquired the same results (Fig. 2). These results show that caveolae restrict the release of TFV virions. As well known, antiviral activity is shown in human cells when the release of enveloped virus particles is inhibited. Up to now, BST-2 (bone marrow stromal cell antigen 2, CD317, Tetherin) is the only one host protein has been discovered to function as the factor to restrict the virus release^41^. BST-2, a membrane protein localized in lipid rafts, inhibits diverse families of enveloped viruses release^42^,^43^. In the case of HIV, the prevention of the release of HIV virions in the absence of Vpu is associated with nascent virions accumulation along the plasma membrane and within clathrin-coated endosomes^44^,^45^. HIV Vpu protein down-regulates BST-2 from the cell surface by interacting with it to antagonize the inhibition^46^,^47^. Our findings suggested that caveolae were possibly another restriction factor of virus release.

The mechanism of BST-2 restrict HIV release was suggested^46^. BST-2 is positioned to directly retain nascent HIV virions on the plasma membrane of infected cells and incorporated into virions. BST-2 restrict HIV release mediated directly by virion-associated and cell-surface-associated BST-2 molecules interact via their ectodomains^46^. In our study, caveolin-1 protein’s incorporation into TFV virions (Fig. 5B), along with the other main structural proteins of caveolae (caveolin-2, cavin-1, and cavin-2) (Fig. 5B), was validated by western blot and immunoelectron microscopy (Fig. 5E). Caveolae-associated proteins (β-actin and filamin) were also detected in purified TFV virions (Fig. 5C). The caveolae-associated proteins were incorporated into TFV virions, but other lipid raft structural proteins, clathrin and flotillin, were not detected in TFV virions (Fig. 5C). We also found that depolymerized caveolae using MβCD affected the released virions in terms of incorporation of caveolar components. Caveolin-1 can be incorporated but no other caveolae associated proteins such as caveolin-2 and cavin-1 (Fig. 5D). Those results implied that the incorporations of caveolae associated proteins were related to the colocalization of TFV and caveolae.

To explain how the caveolae-associated proteins were incorporated into the virions, the main structural protein of caveolae, caveolin-1, was investigated. We determined whether the exogenous fusion caveolin-1 protein could be incorporated into the TFV mature virion. The results showed that caveolin-1-flag can incorporated in the virion, but GFP-flag or Glut-1-flag or flotillin-1-flag protein wasn’t, thereby suggesting that caveolin-1 is not just attached non-specifically to the external or that this protein is perhaps derived from microvesicle or cell debris that was co-purified with the virus (Fig. 6). Furthermore, the interaction of caveolin-1 and TFV MCP were detected using Co-IP/IP and IFA (Fig. 4). Caveolin-1 is the major structure protein of caveolae, like a Scaffold for caveolae to recruit other proteins to this plasma membrane domain. Caveolin-1 has been shown to interact directly with caveolin-2, cavin-1, and cavin-2^48^,^49^,^50^. Thus, we speculate that the caveolae-associated proteins incorporated into TFV virion might be obligated to participate in the interaction between caveolin-1 and TFV MCP at the late stage of infection. To investigate the location of the caveolae-associated proteins within the virion, the virions were subjected to different combinations of proteolytic and/or detergent treatments from the envelope to the capsids. From the inside out, Ranavirus consists of an inner DNA/protein core, an internal limiting membrane, a viral capsid, and an outer viral envelope^29^. Caveolae-associated proteins, caveolin-1, caveolin-2, cavin-1, and cavin-2, were present in the envelope and on the capsid of TFV virions (Fig. 3). Thus, the location of caveolae-associated proteins the further proved these proteins were selectively incorporated.

**Figure 7:**
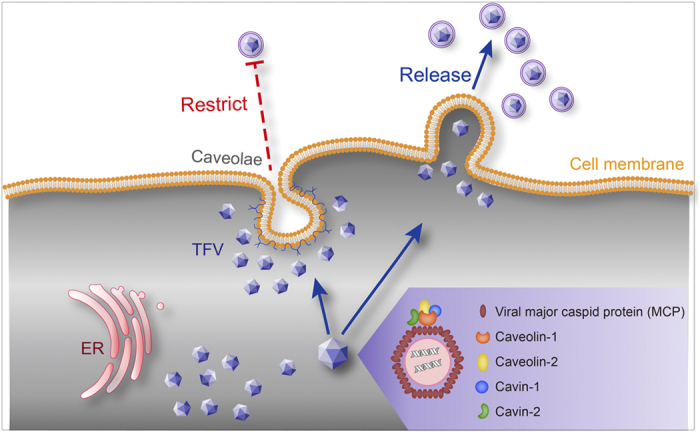
Potential Models of caveolae restricted release of TFV. Caveolae associated proteins incorporated into TFV and located in viral capsid of TFV and caveolae restricted release of TFV.

Our results showed that caveolae restrict the TFV virion release. The potential mechanism of the restriction may be through the interaction of caveolin-1 incorporate in the TFV nascent virion and the proteins in the caveolae (Fig. 7). We found that caveolae-associated proteins can be incorporated in the TFV. However the function of those proteins and detail mechanism of caveolae restrict virus release would need further research.

## Materials and Methods

### Cells and virus

HepG2 cells (ATCC HB8065) and HeLa cells (ATCC CCL-2) were cultured as a monolayer at 37 °C in complete Dulbecco’s modified Eagle’s medium supplemented with 10% fetal bovine serum. TFV was originally isolated from diseased tiger frog (*R. tigrina rugulosa*) tadpoles in Nanhai, Guangdong, China, and was maintained in our laboratory. TFV was grown in fathead minnow cells. Viral infection, stock propagation, and virus titer assay have been described previously^32^.

### Antibodies and reagents

Mouse polyclonal serum against TFV MCP antibody was prepared, as reported previously^51^. MβCD, nystatin, and antibodies specific for cavelin-1, myc-tag, and Flag-tag, were obtained from Sigma-Aldrich (St. Louis, MO, USA). Antibodies specific for caveolin-2, phosphor-caveolin-1 (pTyr14), cavin-2 (SDPR),clathrin, flotillin-1, CD63, Glut-1, CD98, and alpha 1 Sodium Potassium ATPase were purchased from Abcam Co. (Cambridge, UK). Antibodies specific for cavin-1 (PTRF) (N-terminal) and filamin A (C-terminal) were purchased from Epitomics (CA, USA).

### Virus purification and Western blot analysis

Confluent monolayers of HepG2 cells were infected with TFV with a multiplicity of infection (MOI) of 10 and incubated at 27 °C. At 6 d postinfection, the supernatant was harvested before the cells detached, and cell debris was removed by low-speed centrifugation. Virus purification by sedimentation in sucrose density gradients was done as described previously^40^. Protein concentrations of the purified virus stocks were determined using the DC protein assay kit from Bio-Rad (California USA). The purified sample (20 μg) was boiled in sodium dodecyl sulfate (SDS) loading buffer and analyzed using SDS-PAGE on 12% or 15% polyacrylamide gel. Western blot analysis was performed as described previously^52^.

### Plasmid construction and transient transfection

Recombinant DNA techniques were performed according to standard procedures. Human caveolin-1 DNA sequence from HepG2 cells (GenBank Accession No. NG 012051) was cloned into pFlag-CMV4 to generate Flag-hcaveolin-1. The TFV MCP DNA sequence from a TFV stock (GenBank Accession No. AF389451.1) was cloned into pCMV-myc to generate myc-TFV-MCP. The transient transfection of recombinant DNA plasmids into HeLa cells was performed using Lipofectamine^TM^ 2000 (Invitrogen, Carlsbad, CA, USA) according to the instructions of the manufacturer.

### Co-immunopreciptitaion (Co-IP)

HeLa cells were grown in 6 cm dishes and transfected with plasmids. At 24 h post-transfection, cells were lysed with ice-cold lysis buffer containing 10 mM Tris-HCl pH 7.5, 0.4 M NaCl, 1% NP-40, 0.4% Triton X-100, 0.2% sodium deoxycholate, 1 mM EDTA, and protease inhibitors (Calbiochem, USA) for 30 min. Cellular debris were removed by centrifugation at 12,000 g for 15 min at 4 °C. The lysates were immunoprecipitated with antibodies and subsequently adsorbed onto Pierce™ Protein A/G Magnetic Beads (Rockford, USA). Lysates were then collected by centrifugation and washed extensively with 1 ml of washing buffer (10 mM Tris-HCl, pH 7.5, 0.2 M NaCl, and 1 mM EDTA). Immunoprecipitated proteins were solubilized by boiling in alkaline SDS loading buffer and were subjected to SDS-polyacrylamide gel electrophoresis (PAGE) before analysis by immunoblotting as described previously^52^.

### Isolation of caveolae-rich membrane fractions from HepG2 cells

Isolation of caveolae-rich membrane fractions from TFV-infected HepG2 cells in sucrose density gradients was performed. TFV-infected HepG2 cells were washed twice with Ca^2+^- and Mg^2+^-deficient phosphate-buffered saline and scraped in 1 ml of ice-cold lysis buffer containing 1% Triton X-100, 25 mM morpholineethanesulfonic acid (pH 6.5), 150 mM NaCl, 5 mM EDTA, and protease inhibitors as described previously^36^,^37^. After homogenization, cell extracts were adjusted to 40% sucrose by mixing with an equal volume of the lysis buffer containing 90% (w/v) sucrose (without Triton X-100 and protease inhibitors). Subsequently, cell extracts were placed at the bottom of a 12 ml ultracentrifuge tube. A discontinuous gradient was formed above the lysate by adding 6 ml of 30% sucrose solution and 4 ml of 5% sucrose solution. Samples were centrifuged at 190,000 g for 16 to 20 h at 4 °C. Fractions (1 ml) were collected from the top of the gradient. Aliquots of each sucrose density gradient fraction were resolved by SDS-PAGE and analyzed by immunoblotting.

### Immunofluorescence assay (IFA)

HepG2 cells were grown at low density on glass coverslips. Cells were infected with TFV at an MOI of 10. At 72 h postinfection, cells were fixed for 15 min with 4% formaldehyde in PBS at room temperature. Nonspecific binding was blocked by 30 min incubation in 5% normal goat serum. TFV MCP and caveolin-1 were detected by simultaneously staining cells with specific antibodies diluted in PBS. Cells were then washed, and antibody binding was detected using host-specific Alex Fluor-conjugated secondary IgGs (Invitrogen, Carlsbad, CA, USA) as described previously. The coverslips were then washed several times with phosphate-buffered saline with Tween 20 and incubated with Hochest 33342 (Invitrogen, Carlsbad, CA, USA). The samples were examined under a confocal microscope (Zeiss LSM510, Germany). Mock-infected cells were similarly stained as controls.

### Drug treatments

Drug treatments of HepG2 cells at late phase postinfection were performed. HepG2 cells were infected with TFV at an MOI of 10 and were treated with MβCD or nystatin at 60 h postinfection, control cells were incubated in medium with the corresponding solvent.

### PCR analysis TFV genome

TFV genome was determined using two TFV genes, TFV *mcp* gene *orf096R* and TFV gene *orf031L*, located at different region of TFV genome. The primer of TFV *mcp* gene *orf096R* (forward primer 5′-ATGTCTTCTGTAACTGGTTC-3′) and (reverse primer 5′-TTACAAGATTGGGAATCCCATC-3′) were used to amplify a region from 95938 to 97329 of the TFV genome (GenBank Accession No. AF389451.1). the primer of TFV *orf031L*, forward (forward primer 5′-ATGCGTTGTATGCGCTCCGG -3′) and reverse (reverse primer 5′-TCAAGTCCTCGGAGACGTGA-3′) were used to amplify a region from 3582 to 36122 of the TFV genome (GenBank Accession No. AF389451.1).

### Absolute quantitative real-time PCR analysis

TFV genome was determined using a LightCycler 480 instrument (Roche, Germany). Absolute qPCR was performed using TFV *mcp* gene *orf096R* forward (forward primer 5′- TCGCTGGTGGAGCCCTGGTA-3′) and reverse (reverse primer 5′- GGCGTTGGTCAGTCTGCCGTA -3′) primers. The primers were used to amplify a region from 97034 to 97163 of the TFV genome (GenBank Accession No. AF389451.1). Plasmid pCMV-myc, which contains TFV *orf096R*, was used as the internal standard. The internal standard plasmid was serially diluted by 10-folds to generate a standard curve of absolute qPCR. DNA from the supernatant of infected cells was extracted using PureLink™ Viral RNA/DNA Mini Kit (Invitrogen, Carlsbad, CA, USA). The extracted DNA template and the internal standard plasmid were subjected to absolute qPCR. The PCR reaction mixture (10 μl) contained 5 μl 2 × SYBR Premix Ex Taq (TaKaRa, China), 1 μl DNA template, 0.2 μl of 10 μM primers, and 3.6 μl H_2_O. The absolute qPCR conditions were as follows: one cycle at 95 °C for 10 s, 40 cycles of 5 s at 95 °C, 40 s at 60 °C, and 1 s at 72 °C. Absolute qPCR was performed at three replicates per sample.

### TCID_50_ assay

Before infection, prepare 96-well dishes by seeding with cells. Make dilutions at 10^−1^–10^−10^ of the original virus sample in culture medium. Add 0.1 ml of virus dilution to 10 wells for that dilution while add 0.1 ml of culture medium as negative control. Place the dishes at 27 °C. Record the number of positive and negative wells. Calculate the TCID_50_ using Karber method^53^.

### Immunogold labeling

The purified viral particles were absorbed onto Formvar-coated, carbon-stabilized 200-mesh nickel grids. After semi-drying for 15 min, the grids were blocked with 3% bovine serum albumin for 30 min at 37 °C. The grids were rinsed with PBS and then incubated in antiserum against caveolin-1 or the thioredoxin control (1:100 dilution) for 1 h at 37 °C. After washing with PBS, the grids were incubated with goat anti-rabbit IgG conjugated with 10 nm colloid gold (Sigma, USA) for 1 h at 37 °C. The grids were then washed with distilled water and negatively stained with 2% phosphotungstic acid. The viral envelope was removed with 1% (v/v) alkyl phenoxy polythoxy enthanol (Triton X-100) as described previously. The specimens were examined under a transmission electron microscope (JEM-100CX II, Japan).

### Knockdown of caveolin-1 by siRNA

siRNA sequence (5′- GGGACAUCUCUACACCGUUCC-3′)^54^ for caveolin-1 and control siRNA (NC) (Guangzhou Ribobio Co., Ltd) with no homology with caveolin-1 mRNA were tranfected into HepG2 cells using Lipofectamine^TM^ 2000 according to the instructions of the manufacturer. The transfected cells were collected and analyzed via Western blot after 60 h.

### Statistical analysis

qPCR was carried out in three replicates per sample. Data were analyzed by using Roche Abs Quant/2nd Derivative Max, followed by an unpaired-sample t test, to determine the statistical significance between controls and the experimental groups. Statistical significance was accepted at a p value of <0.05. The data are expressed as means ± standard deviations

## Additional Information

**How to cite this article**: He, J. *et al.* Caveolae Restrict Tiger Frog Virus Release in HepG2 cells and Caveolae-Associated Proteins Incorporated into Virus Particles. *Sci. Rep.* **6**, 21663; doi: 10.1038/srep21663 (2016).
